# Reweighting of Sensory Inputs to Control Quiet Standing in Children from 7 to 11 and in Adults

**DOI:** 10.1371/journal.pone.0019697

**Published:** 2011-05-09

**Authors:** Rémy Cuisinier, Isabelle Olivier, Marianne Vaugoyeau, Vincent Nougier, Christine Assaiante

**Affiliations:** 1 UJF-Grenoble 1/CNRS/TIMC-IMAG UMR 5525, Grenoble, France; 2 UMR CNRS 61 49 Groupe “Développement et Pathologie de l'Action”, Université de Provence, Marseille, France; The University of Western Ontario, Canada

## Abstract

How sensory organization for postural control matures in children is not clear at this time. The present study examined, in children aged 7 to 11 and in adults, the postural control modifications in quiet standing when somatosensory inputs from the ankle were disturbed. Since the reweighting of sensory inputs is not mature before 10, we hypothesized that postural stability was more affected in children than in adults when somatosensory inputs were altered and that this postural instability decreased as age increased during childhood. 37 children aged 7 to 11 years and 9 adults participated in the experiments. The postural task was a semi-tandem position with the right foot in front of the left one. Postural performance was measured by means of a force platform. Two experimental conditions were presented to the participants to maintain quiet standing: With or without altered somatosensory inputs (i.e., with or without ankles vibration). Results showed that postural stability -and thus how the reweighting process of the visual/somatosensory inputs matured- increased non-monotonically between 7 years of age and adult age: There was a linear improvement of postural stability from 7 to 10, followed by a more steady behaviour between 10 and 11 and then postural stability increased to reach the adults' level of performance.

## Introduction

Postural control is based on three distinct processes which develop through childhood: (1) a sensory organizational process, in which one or more of the orientation senses (visual, somatosensory and vestibular) are involved and integrated within the Central Nervous System (CNS) [1]; (2) a motor adjustment process, involved in executing coordinated and properly scaled sensorimotor responses [2]; and (3) an internal representation of body scheme that slowly matures during childhood [3], [4]. Both children and adults make use of visual, vestibular and proprioceptive information to control their body posture, but the respective contribution of these sensory inputs varies during ontogenesis [5].

Of the three sensory systems governing postural control, proprioceptive inputs are thought to have the greatest influence in the detection of body sway [6]. Indeed, many developmental studies reported the importance of the proprioceptive system for postural control in children [7]. Investigation of the influence of the sensory systems on postural control in quiet standing in children reported that the somatosensory system is fully developed at 3–4 years [1] or no later than the age of 6 [8]. Nevertheless, from tendinous vibration studies in children from 7 to 15 years of age, various authors reported that children show a delay in the maturation of the integration of the proprioceptive cues to improve postural control [9].

Detection of visual movement allows body stabilization. This coupling between visual perception and action has been reported efficient in newborn babies to generate postural activity at the neck level in response to the visual flow produced by a moving room [10]. In children, it is well established that visual cues play a prominent role in balance control in postural and locomotor tasks [11]. In a recent study, [12] using computerized dynamic posturography in children from 6 to 14 years showed that children had lower equilibrium scores than young adults, especially when visual information was not available or was incorrect. When vision is available this sensory input seems to be predominant for controlling posture during babyhood [10] and childhood [8].

Current data using several tests (Sensory Organization Test (SOT), Motor Control Test (MCT) or Adaptation Test (ADT)) or removed and/or altered sensory conditions are sometimes conflicting regarding the influence of the somatosensory and visual afferent systems on postural control in children. Using a movable platform and visual surround, [13] reported that children younger than 7 years and 6 months could not avoid the influence of sensory inputs providing inappropriate orientation information [11]. showed with development a shift from a visual dependence to a more adult-like dependence with a combination of ankle joint and visual inputs for controlling posture when children were placed on a movable platform capable of antero-posterior displacements or dorsi-plantar flexing rotations of the ankle joint. This shift occurred around 4 to 6 years of age and reached the adult form in 7- to 10-years-old children. Specifically, these children seemed capable to resolve inter-sensory conflicts as adults do.

In contrast, various authors found that optimal stance stability was reached at the age of 15 [9]. Measuring postural responses to support surface displacements [9], showed that children younger than 15 exhibited an increased postural sway as compared to adults when all sensory information was available and accurate. In all conditions with altered somatosensory inputs, postural sway was more pronounced [14]. reported that adults and children aged 7 to 12 have a similar ability to use dynamic visual cues for postural control, whereas 7- to 12-years-old children do not use somatosensory cues to stabilise posture to the same extent as adults when they stood on a fixed or sway-referenced support surface while viewing full-field optic flow scenes that moved sinusoidally (0.1 and 0.25 Hz) in an anterior-posterior direction.

Moreover [15], investigated postural control in children and adults standing upright and lightly contacting the fingertip to a rigid metal plate that moved rhythmically at different frequencies. Light touch to the moving contact surface induced postural sway in all participants. These authors suggested an undeveloped process of reweighting sensory information from different sources to generate an internal estimate of body orientation at age 6 [16]. recorded postural sway when children were presented with simultaneous small-amplitude somatosensory and visual environmental movements. They concluded that inter-modal reweighting was not observed before 10 and that children did not demonstrate an adult-like sensory reweighting before 12–15 years.

The present study investigated the contribution of the somatosensory inputs to static postural control during childhood. The postural performance of children aged 7 to 11 and adults was analyzed in quiet standing when somatosensory inputs from the ankle were disturbed by the tendon vibration technique while visual inputs remained available. The tendon vibration technique has been extensively used to assess the contribution of somatosensory information to standing postural control [17]. Vibration is obtained by positioning vibrators on specific locations and almost selectively activates the muscle spindle Ia afferent fibers, for which the firing rate is recognized to be interpreted by the CNS as a stretching of that muscle. In other words, introducing vibration induces an alteration of the sensory information that subjects can use for postural control. Even though sensitivity to vibration is subject-dependent, it is mainly dependent on the frequency of vibration which has been shown to induce the main effects at 80 Hz, approximately [18]. In children, a recent study in which two vibrators were fixed on the tendons of the soleus and tibialis anterior muscles, showed that vibration disturbed the ankle somatosensation by distorting the perception of this static joint angle at ages 8, 10 and 12 [19]. We hypothesized that postural stability would be more affected by the alteration of somatosensory inputs in children than in adults and that this postural instability observed in children would decrease during childhood.

## Materials and Methods

### Participants

**Figure 1:**
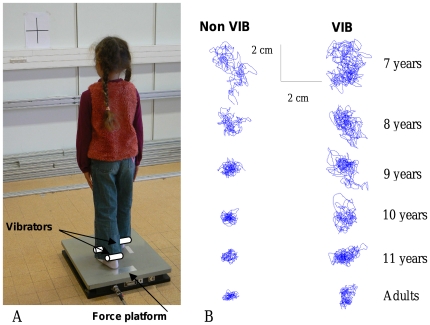
Illustration of A) the experimental set-up and B) the mean stabilograms of representative participants as a function of age and somatosensory condition (left vs. right, non perturbed vs. perturbed somatosensory conditions, respectively).

46 participants, divided into six age groups, participated to the experiment: Eight 7-year-olds (4 girls and 4 boys, *M* = 7.3 years, *SD* = 2.3 months), eight 8-year-olds (3 girls and 5 boys, *M* = 8.2 years, *SD* = 2.4 months), seven 9-year-olds (3 girls and 4 boys, *M* = 9.2 years, *SD* = 4.6 months), six 10-year-olds (4 girls and 2 boys, *M* = 10.1 years, *SD* = 1.7 months), eight 11-year-olds (4 girls and 4 boys, *M* = 11.4 years, *SD* = 3.1 months) and nine adults (2 females and 7 males, *M* = 25.7 years, *SD* = 27 months). Participants were recruited on a voluntary basis from a social middle class, had a normal scholastic level, did not show any known neurological or motor disorders and were right-footed. The “Comité de Protection des Personnes”, zone Sud-Méditerranée I, France, has especially approved this study. In conformity with the Helsinki Convention, informed written consent was obtained from all participants (or parents/guardians) involved in our study. Figure 1 includes an image of a child, seen by back. We confirm that the legal guardian of this child has seen this manuscript and figure and has provided written consent for publication.

### Task and Procedure

Participants stood barefoot with their arms hanging loosely by their sides and their feet placed slightly apart (4 cm on the medio-lateral axis) on marks drawn on the force platform (AMTI®, model OR6-5-1) in a semi-tandem position with the right foot in front of the left one (the tiptoe of the left foot was placed on the same line as the heel of the right one). Vertical force (Fz), frontal and sagittal torques (M_y_ and M_x_, respectively) from the force platform were recorded to compute the displacements of the centre of foot pressure (100 Hz frequency with a 12 bit A/D resolution). Two vibrators (280 g, diameter 4 cm, length 8 cm, and vibration frequency of 80 Hz) were securely strapped over the Achille and tibialis anterior tendons on each foot with elastic bands in order to disturb somatosensory inputs from the ankles.

Participants' task was to sway as little as possible during 30 sec in two altered or non-altered somatosensory conditions (i.e., with or without vibration at the ankles). In these two conditions, they were asked to fixate a picture located 150 cm away from the force platform, at eyes level. For each somatosensory condition, one block of four successive trials was performed. The order of presentation of the two blocks of trials was randomized among participants.

Force platform data were filtered with a 10 Hz low-pass, second order Butterworth filter, this cut-off frequency having been chosen by residuals analysis [20]. Displacements of the centre of foot pressure on the antero-posterior (CoPx) and medio-lateral (CoP_x_) axes were calculated using the following approximation: ΔCoP_x_ = ΔM_y_/F_z_ and ΔCoP_y_ = −Δ M_x_/F_z_ in which ΔM_y_ and ΔM_x_ were a change of the torque with respect to its baseline value (defined as the average value within the time interval from 0 to 30 s). Then, three dependent variables were calculated. (1) The area of the stabilogram reflected a global postural behaviour [21]. Since participants were placed in a semi-tandem position, (2) the mean amplitude and (3) the mean velocity of CoP displacement were analysed in the medio-lateral direction, only. These last two measures have been suggested to represent the amount of activity required to maintain stability [22], providing a more functional approach of postural control.

### Statistical analysis

To explore the sensory integration of somatosensory inputs during the ontogenetic period, a 6 ages (7, 8, 9, 10, 11 years and adults) ×2 somatosensory conditions (with and without vibration) analysis of variance (ANOVA) with repeated measures on the last factor was applied to the mean amplitude and speed of the CoP. The Newman-Keuls Post-hoc test was used whenever necessary. The level of significance was set at p<0.05.

## Results

Analysis of the area of the stabilogram showed a main effect of age *F*_(5,40)_ = 4.03, *P*<0.01, and somatosensory condition, *F*_(1,40)_ = 7.83, *P*<0.01. The area of the stabilogram increased when somatosensory inputs were altered and the Newman-Keuls post hoc tests revealed a significant decrease of the area across the different ages, from 7 to adults (ps<.05, see fig. 1B). No significant interaction was found.

Analysis of mean amplitude replicated and confirmed the results based on the surface. There was a main effect of somatosensory condition, *F*_(1,40)_ = 9.75, *P*<0.01, and a trend for the main effect of age, *F*_(5,40)_ = 2.34, *P*<0.059. Mean amplitude was larger when using vibration at the ankles and slightly diminished across the different ages, from 7 to adults. No significant interaction was found.

**Figure 2:**
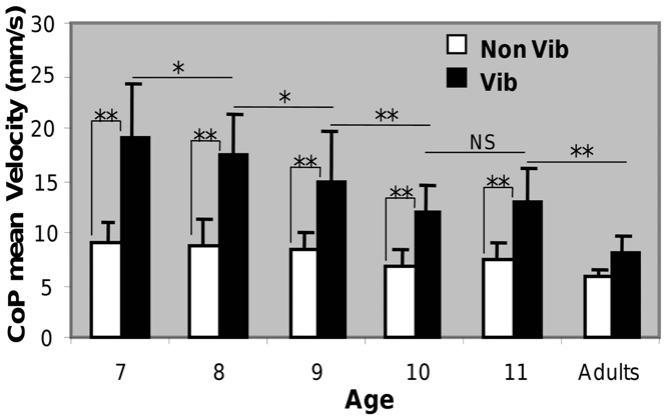
Mean velocity and standard deviation of the medio-lateral displacement of the CoP (mm) according to age and perturbed somatosensory conditions (white bars are without vibration and black ones with vibration). The significant differences are indicated by the asterisks (**p<.01, *p<.05).

As illustrated in Fig. 2, analysis of mean velocity showed main effects of age, *F*_(5,40)_ = 8.09, p<0.0001, somatosensory condition, *F*_(1,40)_ = 262.32, p<0.0001 and a two-way interaction of age x somatosensory condition, *F*_(5,40)_ = 9.25, p<0.0001. The post hoc test revealed that when somatosensory disturbance was not applied, mean velocity did not differ as a function of age (ps>.50). However, when vibration was applied at the ankles, mean velocity significantly increased in 7, 8, 9, 10 and 11 years but not in adults (ps<.001 and p>.05, respectively). Moreover, in the altered condition, post hoc test showed a decrease of mean velocity between 7 and 10 and again between 11 and adults.

## Discussion

The aim of the present study was to investigate, in children aged 7 to 11, the process of visual/somatosensory reweighting to control quiet standing. Our hypothesis was that children would be more affected than adults by the alteration of the somatosensory inputs when vision remained available.

### Age-related differences

In agreement with the literature, the present results showed an improvement of postural stability with age. More specifically, the improvement of postural control during childhood is characterized by a decreasing magnitude [23], and frequency [24] of postural sway. Moreover, the period of 7 to 11 years is considered as a critical period [25] in which an improvement of postural stability is observed, resulting from an integration of the reactive and predictive modes of postural control [26], a more coordinated timing of the muscles involved in postural activity [27], a better integration of visual and vestibular information [1], and the occurrence of an adult-like balance control strategy characterized by a head-stabilization-in space strategy, associated with an articulated operation of the head-trunk unit [5].

### Effects of the alteration of somatosensory inputs on postural sway according to age

The present results showed that when a somatosensory perturbation was applied to the ankles in children aged 7 to 11 years, postural control was affected (i.e., mean CoP velocity increased) whereas it was not the case in adults. As suggested by various authors [15], [16], the alteration of somatosensory inputs was not totally compensated for by the predominant use of vision before the adult age because the inter-modal reweighting process was fully mature after 10 years of age, only. This explanation also suggested that a complete compensation was not possible because the capacity and/or quality of the reweighting process of visual/somatosensory inputs were also maturing from 7 to 10.

When focusing on the different ages within the children, the present results showed that the increase of mean CoP velocity following ankles vibration was smaller with increasing age. More precisely, the magnitude of this effect decreased between 7 and 10 and remained unchanged between 10 and 11. This result suggested that the sensory reweighting in which one or more of the orientation senses (visual, somatosensory and vestibular) are involved and integrated within the CNS is still maturating non linearly between 7 and 11 [1]. In accordance with [9] study (1990) in which children younger than 15 years showed more difficulties with altered somatosensory cues than older subjects, our findings suggested a non-monotonic development of postural control characterised by a linear improvement of the use of visual inputs and/or of the reweighing process of visual/somatosensory inputs from 7 to 10, followed by a more steady behaviour between 10 and 11 and a further improvement until adult age. Finally, the present results supported recent findings suggesting that children do not demonstrate an adult-like use of sensory information prior to the age of 12 [8].

### Conclusion

In conclusion, this study confirmed the existence of age-related differences in the reweighting of visual/somatosensory inputs with a maturation reached very late at the adulthood contrary to other processes (e.g., somatosensory system). The reweighting capacity of visual/somatosensory inputs increased non-monotonically from 7 to adult age, with a linear improvement from 7 to 10 followed by a more steady behaviour between 10 to 11 and a final improvement between 11 to adult age. Further investigations will be necessary to 1) attest the evolution of this phenomenon during the adolescence period, and 2) identify the deficits of the reweighting capacity of sensory inputs for postural control in children with neurological disorders (e.g., children with cerebral palsy, children with attentional deficit/hyperactivity disorders or children with hemiplegia).
